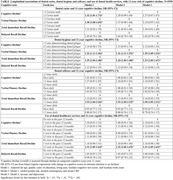# Dental caries, dental hygiene and selfcare, and use of dental health services, and risk of cognitive decline – an 11‐year follow‐up cohort study

**DOI:** 10.1002/alz.088766

**Published:** 2025-01-09

**Authors:** Sam Asher, Anna Liisa Suominen, Ruth Stephen, Tiia Ngandu, Seppo Koskinen, Alina Solomon

**Affiliations:** ^1^ University of Eastern Finland, Kuopio Finland; ^2^ Kuopio University Hospital, Kuopio Finland; ^3^ Finnish Institute for Health and Welfare, Helsinki Finland; ^4^ Karolinska Institutet, Stockholm Sweden; ^5^ Population Health Unit, Finnish Institute for Health and Welfare, Helsinki Finland; ^6^ The Ageing Epidemiology (AGE) Research Unit, School of Public Health, Imperial College London, London United Kingdom

## Abstract

**Background:**

Current evidence links poor oral health, especially tooth loss, with impaired cognition. However, role of underlying causes of tooth loss e.g., dental caries is not known. Moreover, concerning cognition, impact of dental hygiene and selfcare, and use of dental health services is yet to be studied. This study assesses various dental‐health related variables against 11‐year cognitive decline.

**Method:**

Study included ≥30 years old individuals (N = 2958) who participated in the Finnish Health 2000 and its follow‐up Health 2011 Surveys. Dental caries for all teeth (except third molars) and dental hygiene or presence of plaque at 3 teeth/sites were clinically assessed at baseline (2000). Dental selfcare was assessed through daily brushing frequency, and number of visits to dental healthcare professionals in past 12 months indicated use of dental health services. Cognitive assessment at baseline (2000) and follow‐up (2011) was done via cognitive test battery including verbal fluency, immediate and delayed word recall. To assess overall cognition, a composite cognitive score was calculated both at baseline and follow‐up as a sum of individual cognitive tests. For each cognitive test, including composite cognitive score, 11‐year cognitive decline was calculated as difference between scores in 2011 and 2000. Exposure‐Outcome associations were analyzed via binary logistic regression and adjusted for multiple relevant covariates.

**Result:**

After adjustments poor oral hygiene demonstrated by having plaque at ≥2 teeth was associated with decline in verbal fluency and delayed recall. Inadequate dental selfcare demonstrated by brushing <1 times daily and extensive use of dental health services demonstrated by ≥2 visits to different dental healthcare professionals in the past 12 months were both associated with decline in total immediate recall. However, no association for dental caries was observed.

**Conclusion:**

Preliminary results indicated higher risk of cognitive decline in individuals with poor oral hygiene, inadequate oral hygiene practices and recent history of multiple visits to dental healthcare facilities. In light of these findings, future studies into the factors influencing these modifiable patterns are recommended.